# Dyadic Coping of Kidney Transplant Recipients and Their Partners: Sex and Role Differences

**DOI:** 10.3389/fpsyg.2019.00397

**Published:** 2019-02-26

**Authors:** Daria Tkachenko, Laura Franke, Luisa Peters, Mario Schiffer, Tanja Zimmermann

**Affiliations:** ^1^Department of Psychosomatic Medicine and Psychotherapy, Hannover Medical School, Hanover, Germany; ^2^Integrated Research and Treatment Center IFB-Tx, Hannover Medical School, Hanover, Germany; ^3^Department of Nephrology, Hannover Medical School, Hanover, Germany

**Keywords:** kidney transplantation, dyadic coping, couples, relationship quality, stress communication, sex differences, depression, anxiety

## Abstract

**Background:** Coping with stressful health issues – e.g., organ transplantation – can affect interpersonal relationships.

**Objective:** The study examines individual and dyadic coping (DC) in kidney transplant recipients and their partners under consideration of sex and role differences. The Dyadic Coping Inventory allows analyzing partners’ perception of their own DC and also of their partner’s behavior and investigating different perspectives with three discrepancy indexes (similarity, perceived similarity, congruence).

**Methods:** Fifty-six kidney transplant recipients and their partners completed self-report questionnaires (*N* = 112) on DC, depression, anxiety, and relationship satisfaction. The average age of the patients was 58.1 years and of the partners 57.2 years; 64.3% of the patients were male; time since transplantation was on average 9.7 years.

**Results:** (1) Individual and dyadic functioning: In couples with male patients female caregivers showed higher own supportive DC than the males. In couples with female patients, women reported higher own stress communication, supportive DC, total positive DC and total DC as well as depression compared to men. (2) Regarding the discrepancy indexes, in couples with male patients lower levels of similarity in DC reactions of the couple was associated with higher depression of the males as well as higher anxiety of the females. Moreover, lower comparability of the own DC with partner-perception was correlated with higher depression in males. In couples with female patients, higher comparability was associated with higher DC. Higher DC of the males was associated with lower own anxiety and better similarity in DC reactions. Lower levels of similarity of the male spouse showed correlations with higher depression and anxiety of the females. (3) Sex and role differences occurred. No significant differences between male patients and male partners occurred whereas female patients showed higher own stress communication, supportive DC, common DC, total positive DC, total DC and relationship satisfaction compared to female caregivers (role differences). The same differences were found comparing female with male patients. No differences occurred between male and female caregivers (sex differences). (4) Regarding male’s relationship quality, male’s DC total score and similarity index seem to be important predictors in couples with male patients.

**Discussion:** The results demonstrate the relevance of DC in couples with kidney transplantation and show differences between males and females as well as between patients and partners.

## Introduction

Kidney transplantation is the treatment of choice for most patients with end-stage renal disease (Reimer et al., 2002; Pinquart and Sörensen, 2007). However kidney transplantation can be considered as a stressor that impacts the psychological well-being of the patient (Goetzmann et al., 2007). The kidney transplant recipient is permanently exposed to requests in the healthcare context, such as the life-long intake of immunosuppressive medication (Behrend, 2001; Ciesek et al., 2006). Therefore increasing rates of depression, family problems and non-adherence can occur (Laederach-Hofmann et al., 2002; Butler et al., 2004; DiMatteo, 2004; Bunzel et al., 2005). The high rate of non-adherence in kidney transplant recipients can increase the risk of rejection or graft failure (Butler et al., 2004; Denhaerynck et al., 2005; Sellares et al., 2012; Pabst et al., 2015).

The support network of the patient plays an essential role regarding adherence. Relatives and caregivers are important resources providing support to the patient (Lawrence, 1974; Köllner et al., 2004). Most often, spouses are the caregivers of chronically ill patients (Rees et al., 2001). However, not only the patient but also the partner can experience distress when providing care to a chronically ill spouse (Coyne and Smith, 1991; Myaskovsky et al., 2005; Dew et al., 2007; Greif-Higer et al., 2008; Schulz and Sherwood, 2008). Moreover, the significant and often long-term emotional and physical health consequences can negatively impact the relationship quality. In addition, the perceived stress of one partner can influence the other partner’s stress (Frazier et al., 1995; Kadioglu et al., 2012). Depression and anxiety symptoms can occur and negatively impact relationship satisfaction (Arapaslan et al., 2004; Eryilmaz et al., 2005; Noohi et al., 2007). Although social support, especially from the spouse, can be regarded as important for the kidney transplant recipient, also the partners themselves are affected by the disease, which can lead to distress and result in a poorer relationship functioning.

Because the kidney transplantation results in high levels of stress for both, the patient and the spouse, the transplantation can be considered as a challenge for the relationship. Partners within a dyad must be seen as an interdependent whole in which each influences the other (Fife et al., 2013). Hence, to analyze the influence of kidney transplantation on relationship quality and coping abilities seems to be important. The Dyadic Coping Inventory (DCI) is an adapted and frequently used way of assessing the coping process of couples coping with a disease (Bodenmann, 2008) and based on the Systemic Transactional Model (STM) (Bodenmann, 1997; Bodenmann et al., 2016) which describes the intercourse of a couple when one partner is confronted with a stressor and the other partner supports him/her as well as the common efforts a couple makes to cope with a shared stressor (Donato et al., 2012). The STM postulates the mutual impact of one partner’s daily stress experiences, the specific behavior under stress and the well-being on their partner’s experiences. Thus, stressors – such as a kidney transplantation and its medical treatment – affect directly or indirectly both partners of a dyad. As such, even if the kidney transplantation concerns primarily the patient, the stress reactions and coping affect the partner and could turn into dyadic issues, showing the mutuality of stress. The STM emphasizes this mutuality and interdependence between partners. The stress of one partner also affects the other person, but also the resources of one person can expand the resources of the other person. Especially joint appraisal appears as an important dyadic coping (DC) strategy and is linked to dyadic adjustment (Bodenmann et al., 2016). In couples with chronic diseases dyadic appraisals predicted higher levels of mutual self-disclosure and higher mutual responsiveness (Manne et al., 2014).

According to the STM partners can express their stress verbally and/or non-verbally as well as with implicit or explicit requests for assistance. The DC includes partner-oriented behaviors or couple-oriented behaviors and may be positive or negative (Bodenmann et al., 2016). DC is defined as an interplay between the stress signals one partner perceives, the communication of them to his/her partner and the following reactions of the other partner (Revenson et al., 2005; Donato et al., 2015). At the moment when one partner communicates the stressor in the dyadic system, the stressor becomes a dyadic concern. In this context, both, the coping efforts of one partner to support the other when he/she communicates stress and the efforts of both partners to cope together with a common stressor that affects them simultaneously (i.e., common DC), are considered as “dyadic” coping responses (Donato et al., 2015).

The DCI allows calculating sum scores of different coping levels (self-evaluation, partner evaluation, evaluation as a couple) as well as the discrepancy indexes (Gmelch and Bodenmann, 2007; Osin et al., 2018). The discrepancy indexes may help to relate the perception of the own coping and the view of the partner’s coping behavior (Osin et al., 2018). In previous studies the perceived similarity, as the perception of giving and receiving support to be equilibrated in a partnership, emerged as a predictor for partnership satisfaction and the psychological well-being (Gmelch and Bodenmann, 2007; Bodenmann, 2008). More research is needed to define the role of the other discrepancy indexes, such as the similarity and the congruence index, and to describe other outcomes within the concept of DC.

Little is known so far on determining the role of the discrepancy indexes within mental outcomes as distress, depression or anxiety disorder. Nevertheless studies indicate that especially the difference between the perception of the patient and the partner is of huge importance for social support and psychological well-being when coping with a disease (Badr et al., 2010; Regan et al., 2014; Junghaenel et al., 2017; Osin et al., 2018). Studies with couples coping with breast and prostate cancer showed that negative coping of the partner and common negative coping was associated with higher distress levels and higher psychological burden of the patient, whereas positive common coping showed an association with lower distress (Badr et al., 2010; Regan et al., 2014). Comparing the evaluation of pain severity levels in patients suffering from chronically pain between patient and partner, results showed that partners who overestimated the pain severity of the patients report higher support to the spouse whereas the patients did not report more support perceived from their partners (Junghaenel et al., 2017). This finding could lead to misunderstanding and consequently to higher distress levels on both sides. Another study examined couples in which one has been diagnosed with a hemato-oncological disease in regard to social support (DC) and psychological burden (depression, anxiety disorders). Interestingly, the congruence index of the DCI showed a high agreement between the self-evaluation of the DC of the patient and the partner-evaluation. However, the consensus between the self-evaluation of the partners’ DC and the partner-evaluation was lower. Apparently partners could estimate the DC of the patients better than patients estimate the DC of their partners (Osin et al., 2018). However, role differences – being patient or partner – were not investigated. Discrepancies in perception of the coping between patient and partner could help to detect the reciprocal biased perception of the DC, for example the overestimation of the own coping efforts and the underestimation of the other’s coping. This aspect could also be important for analyzing DC in couples with kidney transplantation and its impacts on psychological well-being of patient and partner. To our knowledge no research on discrepancy indexes in couples in which one partner receives a graft was conducted so far.

Furthermore, sex and role (being patient or caregiver) appear to be important factors influencing coping with a kidney transplantation (Bédard et al., 2005). Little is known so far about differences in coping between males and females. For a long time differences in coping have been analyzed only in cancer samples, affecting predominantly one sex (e.g., breast cancer, prostate cancer), so that a differentiation between sex and role within the coping process was difficult. In cancer research, women reported consistently more distress than men regardless of their role (Pinquart and Sörensen, 2007; Hagedoorn et al., 2008). In transplantation research, Bédard et al. (2005) and Holtzman et al. (2011) showed that female caregivers for patients on waiting list for an organ (e.g., lung, heart, liver, and kidney) experience higher distress and higher levels of depression than male caregivers and male patients. Possible reasons for that might be the higher amount of tasks, more time provided in caregiving and less support from other family members offered to female caregivers than to male caregivers (Yee and Schulz, 2000). Additionally female caregivers report about more negative health impact when caring for male transplant recipients than male caregivers, which is associated with depression (Holtzman et al., 2011). Bédard et al. (2005) found that especially women in the caregiver role for men seem to experience higher distress levels compared with female patients or caregivers for female patients. Caregiving has traditionally been viewed as a female role, so that male caregiver may not only receive more support but also more recognition from outside of the dyad (Stoller, 1992). Another aspect to be mentioned is that women resign more frequently social activities in favor of caregiving tasks, which may contribute to feelings of isolation (Navaie-Waliser et al., 2002). Female caregivers show overprotective behaviors when caring for men, which can be seen as a reflector of caregiver’s overcharge (McPherson and Addington-Hall, 2003). The sex seems to possess an important impact on individual’s perception of role (patient versus caregiver) and also on psychosocial aspects.

The current study investigates (1) individual and dyadic functioning of couples after renal transplantation. (2) Moreover, the present study examines differences in coping behaviors between males and females (sex differences) and between patients and caregivers (role differences). From research so far, we expect more DC in females, especially in female caregivers. Furthermore, female caregivers are more likely to experience depression and anxiety than female patients, male caregivers, or male patients. In addition to the total score and the subscales of the DCI the discrepancy indexes that make the perception of differences in assessing the coping between patient and partner possible will be analyzed. (3) Moreover, the discrepancy indexes could shed light on the detection of the interdependence between patients and spouses as well as different perspectives on own and partner DC. Finally, DC in regard to relationship quality was analyzed (4). A positive association between DC and relationship satisfaction was expected regardless of sex or role differences.

## Materials and Methods

### Participants

Kidney transplant patients and their partners were recruited as part of a cross sectional study carried out in the renal transplant clinic at Hannover Medical School. Eligibility criteria included post-mortem renal transplantation patients who underwent transplantation at least 1 year prior, in a heterosexual relationship for at least 1 year, age older than 18 years and German language competence. During a recruitment period of 10 months, from August 2016 until May 2017, patients were asked by phone approximately 1 week before their follow-up visit about their interest to participate at the study and the possibility to come accompanied by the partner. One hundred and forty-six couples fulfilled these criteria. Ninety couples did not participate at the study (most frequent reasons were no interest or time), so that the final sample consisted of *N* = 56 heterosexual couples (recruitment rate of 38.4%). The study was approved by the ethics committee of Hannover Medical School (No. 3003-2016) and all participants provided their written informed consent.

Patient and partner completed questionnaires separately. Sample characteristics are provided in Table 1. 64.3% (*n* = 36) of the kidney transplant recipients were male. The sample was divided in four different groups: male kidney transplant recipients (*n* = 36) and their female partners (*n* = 36), female kidney transplant recipients (*n* = 20) and their male partners (*n* = 20).

**Table 1:** Sample characteristics (*N* = 56).

Demographic	Recipient (*n* = 56)	Caregiver (*n* = 56)
Age mean (*SD*)	58.1 (11.6)	57.2 (11.5)
Sex *n* (%)		
Male	36 (64.3)	20 (35.7)
Female	20 (35.7)	36 (64.3)
Employment *n* (%)		
Employed (full-time)	13 (23.7)	20 (35.6)
Employed (part-time)	4 (7.2)	15 (26.8)
Unemployed	-	1 (1.8)
Retired	34 (61.9)	16 (28.6)
Other	4 (7.2)	4 (7.2)
Education *n* (%)		
<10 years	23 (41.8)	22 (40.7)
10 years	18 (32.7)	16 (29.6)
>10 years	11 (20.0)	14 (25.9)
other	3 (5.5)	2 (3.7)
Time since transplantation in years mean (*SD*)	9.4 (6.7)	
Relationship status *n* (%)		
Married	49 (87.5)	
Unmarried	7 (12.5)	
Relationship length in years mean (*SD*)	30.8 (14.1)	

The mean age of the male kidney recipients was 59.08 years (*SD* = 11.0, 37–78), of the female transplant recipients 56.40 years (*SD* = 12.6, 35–79), 58.85 years (*SD* = 13.8, 35–85) of the male partners and 56.33 years (*SD* = 10.2, 35–74) of the female partners. Male patients were significant older than their female partners [*t*(35) = 4.278, *p* = 0.000]. Relationship length did not differ between couples with female kidney transplant recipients (*M* = 30.85 years, *SD* = 14.59, 9–56) and couples with male transplant recipients [*M* = 30.64 years, *SD* = 14.0, 2–54; *t*(54) = 0.053, *p* = 0.958]. No differences were found for time since transplantation between male (*M* = 9.46 years, *SD* = 7.04, 1–27) and female kidney transplant recipients [*M* = 9.15 years, *SD* = 6.18, 2–28; *t*(53) = 0.163, *p* = 0.871].

### Measurements

#### Dyadic Coping

The Dyadic Coping Inventory (Bodenmann, 2008), a standardized assessment of DC within couples under conditions of stress, was used to measure DC (Gmelch et al., 2008). The DCI contains 37 items rated on a 5-point Likert scale from 1 (very rarely) to 5 (very often). Patient and partner, both answer questions separately about the own coping perception of oneself (self-evaluation, 15 items), as well as how he/she meets with the other’s coping behavior (partner evaluation, 15 items), about how he/she perceives the coping of their couple (we-evaluation, 5 items) and about the general satisfaction with DC (2 items). The DCI consists of the following nine subscales: Own stress communication (e.g., ‘I let my partner know when I appreciate his/her practical support, advice, or help’), own supportive coping (e.g., ‘I show empathy and understanding’), own delegated DC (e.g., ‘I take on things that my partner would normally do in order to help him/her out’), own negative DC (e.g., ‘I blame my partner for not coping well enough with stress’), stress communication of partner (e.g., ‘My partner asks me to do things for him/her when he has too much to do’), supportive coping of partner (e.g., ‘My partner expresses that he/she is on my side’), delegated DC of partner (e.g., ‘When I am too busy, my partner helps me out’), negative DC of partner (e.g., ‘My partner does not take my stress seriously’), common DC (e.g., ‘We try to deal with the problem together and look for concrete solutions’). *Stress communication* tends to seek for partner’s attention and interest in one’s stress experience with asking for problem- or emotion-oriented support. *Supportive DC* should reduce stress by resolving the concrete problem or reduce emotional stress arousal in assisting the others own efforts. *Delegated DC* diminishes stress arousal by relieving the partner. *Common DC* helps sharing negative emotions in an attempt to regulate them jointly. *Negative DC* can be hostile (e.g., blaming, criticizing, sarcasm), ambivalent (support in an unwilling and unmotivated support) or superficial (support with no motivation, no authentic empathy or no real understanding) (Bodenmann et al., 2016).

Thus a total score ranging from 37 to 187 (cut off values <111 DC below average, values >145 DC above average, 111–145 average DC) and two combined scales “total negative DC” (own negative DC plus negative DC of the partner) and “total positive DC” (own positive DC plus positive DC of the partner) can be assessed.

**FIGURE 1:**
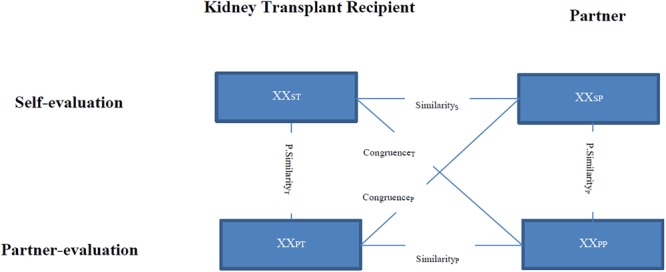
Discrepancy indexes of the Dyadic Coping Inventory (DCI). XX, items about the stress communication, the supportive, delegated, negative dyadic coping (DC). S, self-evaluation (own DC, own supportive coping). P, partner-evaluation (supportive DC of the partner). T, kidney transplant recipient. P, partner. Modified from Bodenmann, 2008. With kind permission by Hogrefe Verlag Berne.

In addition to the total score and the subscales, the DCI allows combining the different perspectives of men and women (self-evaluation and partner-evaluation). These discrepancy indexes, yielding interpersonal congruence for DC strategies were calculated (see Figure 1). The *similarity index* shows how both partners agree on the same subscale and is a measure of similarity of the DC reactions (e.g., “What do I do when my partner is stressed?”). The *perceived similarity index* measures how comparable the own DC (self-evaluation) with the partner-perception is. Items like “What do I do when my partner is stressed?” and “What does my partner do when I am stressed?” are compared. The *congruence index* assesses how both partners consistently experience the DC of the other. Items like “What do I do when my partner is stressed?” of one partner and “What does my partner do when I am stressed?” of the other partner are compared. Lower scores of the indexes are better because they stand for high accordance within a dyad. Cronbach’s alpha in the current sample is 0.92.

#### Depression

The German version of the nine-item Patient Health Questionnaire-Depression Scale (Kroenke et al., 2001) was used. The PHQ-9 is a well validated and widely used depression questionnaire. Participants were asked how often, during the past 2 weeks, they have been burdened, for example with insomnia, and response options were “not at all,” “several days,” “more than half the days,” “nearly every day” scored from 0 to 3, respectively. The total score ranges from 0 to 24. The corresponding severity levels were bordered as none (0–4), mild (5–9), moderate (10–14), moderately severe (15–19), and severe (20–27). Cronbach’s alpha in the current sample is 0.83.

#### Anxiety

The General Anxiety Disorder Screener (GAD-7) is a one-dimensional, self-administered, valid and efficient tool for assessing Generalized Anxiety Disorder and measuring its severity in research and clinical practice (Spitzer et al., 2006). The participant scores the frequency of statements from 0 (“not at all”) to three (“nearly every day”). The total GAD-7 score is computed by addition of the answers to each item. Therefore, the total score ranges from 0 and 21 and may be categorized into four severity groups: minimal (0–4), mild (5–9), moderate (10–14) and serious (14–20). Cronbach’s alpha in the current sample is 0.83.

#### Relationship Satisfaction

Relationship satisfaction was assessed with the German version of the Quality of Marriage Index (QMI-D; Zimmermann et al., 2015). The QMI-D is a six-item questionnaire that uses broadly verbalized statements, such as “We have a good relationship.” The participants indicate their degree of agreement on a scale ranging from one (very strong disagreement) to 7 (very strong agreement) completing five of the six items. The sixth item ranges from 1 (very strong disagreement) to 10 (very strong agreement). The total score ranges from 6 to 45. Cut off values under 34 stand for low partnership quality. Cronbach’s alpha in the current sample is 0.89.

### Statistical Analysis

Statistical analysis was carried out with the IBM SPSS Statistics 25.0 software program. Comparisons of socio-demographic and medical characteristics of the participants were presented in absolute frequencies, percentages, mean values and standard deviations. Multilevel modeling (MLM) with a pairwise dataset was performed to examine the actor and partner effects of DC, as well as of relationship quality, depression and anxiety. MLM can account for inter-dependence within the analyses of couples and is considered one of the best methods to examine effects in the Actor-Partner-Interdependence Model (APIM) (Kenny et al., 2006). *T*-test for independent subgroups were calculated to assess the differences in means for sex (males versus females) and role (patient versus partner). Cohen’s *d* was calculated to indicate the effect size for the comparison between two means. Pearson’s correlations were calculated to assess the link between total score of the DC, discrepancy indexes, depression and anxiety of patients and partners. Multiple regression analysis of relationship quality, as the central outcome parameter of our study was conducted because of sample size only with the subgroup of male kidney transplant recipients (*n* = 36). The applied predictors were different scales of the DCI (DC total score and discrepancy indexes of males and females) and the age of male patients and female partners. Significance level for all analysis was determined to a 5% level.

## Results

### Individual and Dyadic Functioning in Couples After Renal Transplantation

Multi-level modeling was conducted to examine differences in means between men and women within the couple. The analyses were conducted separately for couples with male and female kidney transplant recipients.

#### Couples With Male Kidney Transplant Recipients

In couples with *male kidney transplant recipients* the only significant difference emerged for own supportive behavior with female partners showing significant higher own supportive behavior than the male patients [*M*_♂_ = 11.7, *SD* = 3.2, *M*_♀_ = 12.6, *SD* = 3.1; *t*(35) = -1.26, *p* = 0.02]. No differences regarding the other subscales of the DCI and the discrepancy indexes, relationship quality, depression, and anxiety between male patients and their female partners occurred (see Tables 2, 3).

**Table 2:** Differences in the subscales of the Dyadic Coping Inventory within the couple.

	Male transplant recipient (*n* = 36)		Female partner (*n* = 36)		Difference		
	Mean (*SD*)	Stanine	Mean (*SD*)	Stanine	*t*	*p*	Effect size (*d*)
Own stress communication	11.7 (3.2)	5	12.6 (3.1)	5	–1.26	0.216	0.286
Own supportive coping	18.3 (2.5)	5	19.6 (3.3)	6	–2.38	**0.023**	0.444
Own delegated DC	7.4 (1.5)	5	7.8 (1.8)	6	–1.03	0.311	0.241
Own negative coping	8.0 (2.8)	1	7.3 (2.7)	1	1.25	0.220	–0.255
Common DC	16.4 (3.0)	5	16.9 (3.5)	5	–0.90	0.373	0.153
Total negative DC	15.5 (5.0)		15.2 (5.7)		0.36	0.722	–0.056
Total positive DC	68.9 (7.9)		69.7 (11.5)		–0.51	0.613	0.082

	**Female transplant recipient (*n* = 20)**		**Male partner (*n* = 20)**		**Difference**		
	**Mean (*SD*)**	**Stanine**	**Mean (*SD*)**	**Stanine**	***t***	***p***	**Effect size (*d*)**

Own stress communication	16.0 (2.7)	7	11.9 (4.1)	5	3.76	**0.001**	–1.181
Own supportive coping	21.4 (3.1)	7	18.4 (3.0)	5	3.65	**0.002**	–0.983
Own delegated DC	7.4 (2.2)	5	7.4 (2.2)	5	0.00	1.000	0
Own negative coping	6.9 (3.1)	1	7.1 (3.0)	1	–0.21	0.839	0.066
Common DC	19.5 (3.2)	7	17.7 (3.9)	6	1.84	0.084	–0.505
Total negative DC	13.4 (5.6)		13.7 (5.5)		–0.37	0.713	0.054
Total positive DC	77.1 (9.5)		68.4 (10.5)		3.53	**0.002**	–0.869

*Stanine reference group of the validation sample of Bodenmann (2008): men/women > 50 years, 1 = far below average, 2–3 = below, average, 4–6 = average, 7–8 = above-average, 9 = far above-average. Significant differences in bold. *t* = *t*-test for dependent samples; *SD* = standard deviation.*

**Table 3:** Differences in dyadic coping, relationship satisfaction, depression, and anxiety within the couple.

		Male transplant recipient (*n* = 36)		Female partner (*n* = 36)		Difference	
	Range	Mean	*SD*	Mean	*SD*	*t*	*p*
Total dyadic coping (DCI)	35–175	125.5	11.3	127.3	18.0	–0.69	0.494
Relationship quality (QMI)	6–45	39.7	5.4	38.8	6.4	0.82	0.417
Depression (PHQ-9)	0–27	5	3.4	4.4	3.5	0.89	0.382
Anxiety (GAD-7)	0–21	3.8	3.5	4.3	3.4	–0.66	0.516
Congruence index (DCI)	0–120	12.3	4.4	12.3	4.8	0.03	0.976
Perceived similarity (DCI)	0–120	10.6	4.8	10.9	6	0.24	0.813

		**Self-evaluation**		**Partner-evaluation**			
		***M***	***SD***	***M***	***SD***		

Actual reciprocity (DCI)	0–120	13.3	4.3	12.9	5.6	0.33	0.743

		**Female transplant recipient (*n* = 20)**		**Male partner (*n* = 20)**		**Difference**	
		**Mean**	***SD***	**Mean**	***SD***	***t***	***p***

Total dyadic coping (DCI)	35–175	138.8	17.9	128.2	16.8	3.27	**0.004**
Relationship quality (QMI)	6–45	42.7	2.8	40.1	7.9	1.86	0.079
Depression (PHQ-9)	0–27	5.8	4.7	3.3	2.8	2.24	**0.038**
Anxiety (GAD-7)	0–21	3.6	3.3	3.3	2.4	0.35	0.073
Congruence index (DCI)	0–120	11.8	4.7	13.4	5.5	–1.29	0.218
Perceived similarity (DCI)	0–120	11.3	4.6	11.5	4.9	–0.1	0.923

		**Self-evaluation**		**Partner-evaluation**			
		***M***	***SD***	***M***	***SD***		

Actual reciprocity (DCI)	0–120	15.0	5.3	13.0	6.2	0.33	0.337

*DCI, Dyadic Coping Inventory: cut off < 111 DC below the average, 111–145 average DC, >145 DC above the average; QMI, Quality of Marriage Index (6–45), cut-off < 34 low partnership quality, >34 high partnership quality; PHQ, Patient Health Questionnaire (0–24): scores of 5, 10, 15, 20 represent mild, moderate, moderately severe, and severe depression; GAD, General Anxiety Disorder (0–21): scores of 5, 10, 15 represent a mild, moderate and severe level of anxiety severity. Significant differences in bold.*

#### Couples With Female Kidney Transplant Recipients

In couples with *female kidney transplant recipients*, women showed significant higher total DC than their male spouses [*t*(19) = 3.27, *p* = 0.004, *d* = -0.61] as well as higher depression scores [*t*(19) = 2.24, *p* = 0.038, *d* = -0.65; see Table 2]. Regarding the subscales of the DCI (see Table 1), female kidney transplant recipients compared to their male spouses showed higher own stress communication [*t*(19) = 3.76, *p* = 0.001, *d* = -1.18], more own supportive DC [*t*(18) = 3.65, *p* = 0.002, *d* = -0.98] as well as more total positive DC [*t*(19) = 3.53, *p* = 0.002, *d* = 0.87]. No differences emerged for the DC discrepancy indexes (see Table 2).

#### Relationship Between the Independent Variables

Correlations among the independent variables were tested (see Table 4). In couples with *male transplant recipients*, in particular, significant positive correlations appear between DC of patient and spouse (*r* = 0.48, *p* < 0.01) as well as between relationship satisfaction between patient and spouse (*r* = 0.37, *p* < 0.05). DC of the male patient was positively associated with his relationship satisfaction (*r* = 0.57, *p* < 0.01) as well as the relationship satisfaction of the women (*r* = 0.40, *p* < 0.05). DC of the male patient was negatively associated with the perceived similarity index (*r* = -0.36, *p* < 0.05), meaning that higher DC was associated with higher comparability of own and partner DC and vice versa. DC of the female spouse showed positive correlations with own relationship satisfaction (*r* = 0.63, *p* < 0.01) but not with the relationship satisfaction of the male patient (*r* = 0.16, *p* = 0.34).

**Table 4:** Correlations between dyadic coping, marital satisfaction, depression and general anxiety within the couple.

	DC TX	DC P	QMI TX	QMI P	PHQ TX	PHQ P	GAD TX	GAD P	SimS	SimP	Cong TX	Cong P	PerSi TX	PerSi P
**Male transplant recipient and female partner (*n* = 36)**											
DC TX														
DC P	0.484**													
QMI TX	0.565**	0.162												
QMI P	0.402*	0.626**	0.370*											
PHQ TX	–0.150	0.239	–0.311	0.078										
PHQ P	0.036	–0.80	–0.036	–0.242	0.208									
GAD TX	–0.037	0.088	–0.165	–0.029	0.666**	0.212								
GAD P	–0.049	–0.178	–0.142	–0.128	0.323	0.802**	0.313							
SimS	–0.092	0.081	–0.159	0.166	0.374*	0.220	0.134	0.349*						
SimP	–0.134	–0.264	0.266	–0.015	0.079	–0.030	0.281	0.078	0.376*					
Cong TX	–0.113	–0.192	0.117	–0.021	0.212	–0.160	0.191	–0.120	0.492**	0.650**				
Cong P	–0.278	–0.067	0.092	0.066	0.125	–0.071	0.13	0.104	0.367	0.576**	0.299			
PerSi TX	–0.361*	–0.105	–0.186	0.075	0.458**	0.086	0.325	0.241	0.562**	0.291	0.224	0.3 24		
PerSi P	–0.201	–0.364	–0.077	–0.143	0.261	0.059	0.311	0.276	0.502**	0.446**	0.562	0.359	0.313	
**Female transplant recipient and male partner (*n* = 20)**														
DC TX														
DC P	0.654**													
QMI TX	0.203	0.415												
QMI P	0.108	0.135	0.691**											
PHQ TX	0.015	0.326	0.157	0.148										
PHQ P	–0.342	–0.411	–0.275	–0.090	0.192									
GAD TX	–0.075	0.268	0.157	0.231	0.931**	0.298								
GAD P	–0.475*	–0.513*	–0.043	–0.056	0.119	0.782**	0.137							
SimS	–0.017	–0.158	–0.118	–0.144	–0.182	–0.044	–0.382	–0.086						
SimP	–0.114	–0.631**	–0.032	0.363	–0.516*	0.049	–0.512*	0.084	0.315					
Cong TX	0.088	–0.368	0.039	0.384	–0.286	0.226	–0.207	0.223	0.454	0.683**				
Cong P	0.107	–0.353	–0.450	–0.261	–0.041	0.127	–0.148	0.123	0.714**	0.273	0.548*			
PerSi TX	–0.644**	–0.205	–0.069	0.169	0.460	0.193	0.408	0.193	0.104	0.065	–0.062	–0.113		
PerSi P	0.182	–0.077	–0.050	–0.320	–0.112	–0.054	–0.280	–0.072	0.710**	0.201	0.101	0.536*	–0.310	

*TX, transplant recipient; P, partner; DC, dyadic coping; QMI, Quality of Marriage Index; PHQ, Patient Health Questionnaire; GAD, General Anxiety Disorder; SimS/P, Similarity Index Self/Partner-evaluation; Cong, congruence index; PerSi, perceived similarity index. ^∗^*p* < 0.05; ^∗∗^*p* < 0.01.*

Regarding the discrepancy indexes of the DCI, the similarity index (self-evaluation) showed positive associations with PHQ of the male patient (*r* = 0.37, *p* < 0.05) as well as GAD of the women (*r* = 0.35, *p* < 0.05). Lower levels of similarity in DC reactions of the couple was associated with higher depression of the male patient as well as higher anxiety of the female spouse. Moreover, the perceived similarity of the male patient showed significant positive correlations with his depression score (*r* = 0.46, *p* < 0.01), meaning that lower comparability of the own DC with partner-perception is associated with higher depression in male patients and vice versa.

In couples with *female transplant recipients*, significant positive correlations appear between DC of patient and spouse (*r* = 0.65, *p* < 0.01) as well as between relationship satisfaction between patient and spouse (*r* = 0.69, *p* < 0.01). The DC of the female patient was negatively associated with anxiety of the male partner (*r* = -0.48, *p* < 0.05) as well as with own perceived similarity (*r* = -0.64, *p* < 0.01), meaning that higher comparability is associated with higher DC and vice versa. The DC of the male spouse showed negative correlations with own anxiety (*r* = -0.51, *p* < 0.05) and own similarity index (*r* = -0.63, *p* < 0.01). Higher DC of the males was associated with lower own anxiety and better similarity in DC reactions and vice versa. In addition, lower levels of similarity of the male spouse showed associations with higher depression of the female patient (*r* = -0.52, *p* < 0.05) and higher anxiety of the women (*r* = -0.51, *p* < 0.05) and vice versa (see Table 4).

### Sex and Role Differences

Independent *t*-tests were conducted to analyze differences regarding sex and role. Comparisons were done between male patients and female patients as well as male caregivers and female caregivers (sex differences) and between male patients and male caregivers as well as female patients and female caregivers (role differences).

#### Sex Differences

For *patients*, female transplant recipients compared to male transplant recipients showed significant higher own stress communication [*M*_♂_ = 11.7, *SD* = 3.2, *M*_♀_ = 16.0, *SD* = 2.7; *t*(54) = -5.02, *p* = 0.000, *d* = 1.39], higher own supportive coping [*M*_♂_ = 18.3, *SD* = 2.5, *M*_♀_ = 21.3, *SD* = 3.1; *t*(54) = -3.93, *p* = 0.000, *d* = 1.10], more common DC [*M*_♂_ = 16.4, *SD* = 3.0, *M*_♀_ = 19.5, *SD* = 3.2; *t*(51) = -3.48, *p* = 0.001, *d* = 1.01], as well as more total positive DC [*M*_♂_ = 68.9, *SD* = 7.9, *M*_♀_ = 77.1, *SD* = 9.5; *t*(54) = -3.47, *p* = 0.001, *d* = 0.97] and overall DC [*M*_♂_ = 125.5, *SD* = 11.3, *M*_♀_ = 138.8, *SD* = 17.9; *t*(27.6) = -3.00, *p* = 0.01, *d* = 0.95]. Moreover, differences in relationship satisfaction were found [*M*_♂_ = 39.7, *SD* = 5.4, *M*_♀_ = 42.7, *SD* = 2.8; *t*(53.7) = -2.77, *p* = 0.01]. No differences occurred regarding depression [*M*_♂_ = 5.0, *SD* = 3.4, *M*_♀_ = 5.8, *SD* = 4.7; *t*(54) = -0.73, *p* = 0.47] or anxiety [*M*_♂_ = 3.8, *SD* = 3.5, *M*_♀_ = 3.6, *SD* = 3.3; *t*(54) = 0.30, *p* = 0.77].

In the *caregiving role*, no significant differences between male and female spouses were found in terms of DC as well as for depression [*M*_♂_ = 3.3, *SD* = 2.8, *M*_♀_ = 4.4, *SD* = 3.8; *t*(54) = -1.17, *p* = 0.25], anxiety [*M*_♂_ = 3.3, *SD* = 2.4, *M*_♀_ = 4.3, *SD* = 3.4; *t*(54) = -1.18, *p* = 0.24] or relationship satisfaction [*M*_♂_ = 40.1, *SD* = 7.8, *M*_♀_ = 38.7, *SD* = 6.4; *t*(54) = 0.70, *p* = 0.49] (see Tables 2, 3).

#### Role Differences

For *women*, female transplant recipients compared to female caregivers showed significant higher own stress communication [*M*_patients_ = 16.0, *SD* = 2.7, *M*_caregivers_ = 12.6, *SD* = 3.1; *t*(54) = 4.05, *p* = 0.000, *d* = -1.15], higher common DC [*M*_patients_ = 19.5, *SD* = 3.2, *M*_caregivers_ = 16.9, *SD* = 3.5; *t*(51) = 2.60, *p* = 0.012, *d* = -0.77], more positive DC [*M*_patients_ = 77.1, *SD* = 9.5, *M*_caregivers_ = 69.7, *SD* = 11.5; *t*(54) = 2.45, *p* = 0.018, *d* = -0.69] and overall DC [*M*_patients_ = 138.8, *SD* = 17.9, *M*_caregivers_ = 127.3, *SD* = 18.0; *t*(54) = 2.28, *p* = 0.05, *d* = -0.64] as well as higher relationship satisfaction [*M*_patients_ = 42.7, *SD* = 2.8, *M*_caregivers_ = 38.8, *SD* = 6.4; *t*(51.6) = 3.20, *p* = 0.002]. No significant differences occurred for depression [*M*_patient_ = 5.8, *SD* = 4.7, *M*_caregiver_ = 4.4, *SD* = 3.4; *t*(54) = 1.30, *p* = 0.20] or anxiety [*M*_patient_ = 3.6, *SD* = 3.3, *M*_caregiver_ = 4.3, *SD* = 3.4; *t*(54) = -0.77, *p* = 0.45].

For *males*, no significant differences were found between male transplant recipients and male spouses in terms of DC as well as for depression [*M*_patient_ = 5.0, *SD* = 3.4, *M*_caregiver_ = 3.3, *SD* = 2.8; *t*(54) = 1.90, *p* = 0.06] or anxiety (*M*_patient_ = 3.8, *SD* = 3.5, *M*_partner_ = 3.3, *SD* = 2.4; *t*(54) = 0.66, *p* = 0.51] (see Tables 2, 3).

### Association Between DC and Age With Relationship Satisfaction in Couples With Male Kidney Transplant Recipients

Due to the small sample size of couples with female transplant recipients (*n* = 20), the analysis was only conducted with couples with male patients (*n* = 36). A multiple regression analysis with the relationship quality of male kidney transplant recipients as dependent variable was calculated to assess the impact of DCI (total score, discrepancy indexes) and age of the patients and their partners (Table 5). A significant regression model with 67.1% of explained variance emerged [*F*(9,25) = 2.30, *p* = 0.043, *R^2^*= 0.67]. As significant predictors of male’s relationship satisfaction occurred the DCI total score of male kidney transplant recipients (β = 0.75, *p* = 0.000) and the similarity index of the self-evaluation of male kidney transplant recipients (β = -0.49, *p* = 0.016) as actor effects. No significant partner effects on male’s relationship satisfaction were found.

**Table 5:** Summary of regression analysis for variables predicting relationship satisfaction (QMI) in male transplant recipients (*n* = 36).

	*B*	*SE B*	β	*p*
Males total DC	0.357	0.080	0.747	**0.000**
Males similarity index-self-evaluation (DCI)	–0.623	0.242	–0.491	**0.016**
Females congruence index (DCI)	0.416	0.205	0.365	0.053
Males similarity index – partner-evaluation (DCI)	0.198	0.201	0.203	0.333
Males congruence index (DCI)	0.227	0.240	0.185	0.355
Males age	0.092	0.067	0.185	0.181
Males perceived similarity (DCI)	0.143	0.187	0.125	0.454
Females perceived similarity (DCI)	–0.090	0.161	–0.098	0.583
Females total DC	–0.021	0.051	–0.070	0.680

*DC, dyadic coping; DCI, Dyadic Coping Inventory; QMI, Quality of Marriage Index. Significant differences in bold.*

## Discussion

The current study examined DC of 56 couples in which one partner is a post-mortem kidney transplant recipient and investigated the relationship between DC, relationship satisfaction and depressive and anxiety symptoms taking sex and role differences into account. The DC analysis involved calculating the discrepancy indexes (similarity index, perceived similarity index, congruence index). Finally, the association of these indexes with relationship quality, depression and anxiety were examined.

### Individual and Dyadic Functioning in Couples After Renal Transplantation Under Consideration of Sex and Role Differences

Female kidney transplant recipients emerged as the group with significantly higher levels of depression compared to their male partners. In addition, female patients showed higher DC compared to their male partners, female caregivers and male patients. One possible explanation could be that women experience higher distress levels compared to men when confronted with a disease regardless of the specific diagnosis and whether they are in the patient’s or the caregiver’s role (Holtzman et al., 2011; Fife et al., 2013). Studies on kidney transplantation confirm these aspects: Women with end-stage renal disease react with an increase of immunologic parameters, such as interleukin-1, to the social environment, whereas no associations were found in men with end-stage renal disease (Kimmel et al., 2000; Kimmel, 2001; Kimmel and Patel, 2003). These differences between female and male patients could indicate a more intensive reaction of the female organism toward stress when being confronted with kidney diseases and accordingly the higher need of coping. The interaction between immunologic parameters, depression and DC should be investigated in more detail in future studies.

Another aspect to be considered is the different self-perception of men and women. Kimmel (2001) reported different role expectations: men are supposed to be independent, so that after kidney transplantation they regain quality of life, whereas women are characterized by being emotional, dependent and be physical beauty. Women seem to profit less from the kidney transplantation in regard to quality of life than men and experience more stress (Johnson et al., 1998; Kimmel, 2001).

To better understand the situation of a dyad dealing with kidney transplantation, the time before the transplantation should also be considered. The link between negative affect before and after kidney transplantation has been shown by Szeifert et al. (2010). Dialysis can be seen as a state of prolonged stress for the whole family (Pomaki et al., 2011). In order not to burden other family members, women tend to overcharge themselves, so that finally they might experience higher levels of distress and depression than men (Kimmel and Patel, 2003). Patients who underwent dialysis treatment frequently experience multiple losses, such as the loss of the original role within the family and the dyad, the loss of cognitive abilities and physical power (Kimmel, 2001, 2002). The loss of the original role within the family means a repositioning within the dyad, which is not automatically nullified after kidney transplantation (Pomaki et al., 2011). The higher level of depression of female kidney recipients in our sample occurred only in comparison to their partner (within the couple) and is not explainable through role (patient versus caregiver) or sex (male versus female). The high level of DC occurred in comparison to all subgroups (male partners, female patients, and male patients). That women use the dyadic system more extensively to cope than men is in line with findings of Acitelli and Antonucci (1994).

Some studies indicate that especially female caregivers caring for men are at great risk for developing high distress levels within a dyad (Yee and Schulz, 2000; Holtzman et al., 2011). Possible explanations are that women receive less support from outside of the dyad (other family members, friends), receive less recognition, provide more time to caregiving, and comply more tasks in number (Yee and Schulz, 2000; Kim et al., 2006; Fife et al., 2013). Additionally, women frequently are burdened. They are juggling house work, children and work outside of the home (Stoller, 1992; Fife et al., 2013). Our results are not in line with these findings (female caregivers did not show high scores of depression, anxiety or DC), but hints at why women in general might be more affected and feel the need to cope more when dealing with a disease.

The question why especially female kidney transplant recipients compared to their partner report higher levels of depression might be explained by the behavior of male caregivers: Men in the caregiver perspective of the spouse tend to use negative expressiveness and overprotectiveness as coping strategies (Thompson and Sobolew-Shubin, 1993; Pomaki et al., 2011). The negative expressiveness has been shown to be associated with mortality in female but not in men end-stage renal disease patients (Pomaki et al., 2011). Additionally, women often perceive themselves as a burden and feel overprotected as a patient (McPherson et al., 2010; Holtzman et al., 2011). Both elements, the perceived burden and overprotectiveness, are described as risk factors for developing distress and depression (McPherson et al., 2010). According to the Equity Theory of Wilson et al. (2005), a partnership has to be in balance of giving and receiving support. Chronic illnesses provide the impression of not being able to establish this equilibrium to the patient, so that consequently feelings of worthlessness and depression might develop (McPherson et al., 2010). Overprotectiveness could transmit the feeling of being able to do less than the health estate permits. Coping strategies of male caregivers might be the reason why women experience higher levels of depression and distress in the patient role compared to the partners in our sample. Whether men use negative expressiveness or overprotectiveness more often when caring for their partners within the context of kidney transplantation should be investigated in further studies.

Our results suggest that female kidney transplant recipients are at risk for developing depression with high needs for coping within the couple. Possible explanations for these circumstances could be somatic aspects of females, role and perception of women in our society, as well as male partners using unfavorable coping strategies as a caregiver. A further analysis of important risk factors for depression after kidney transplantation and the impact of DC is warranted.

### Impact of Dyadic Coping and Its Discrepancy Indexes on Relationship Satisfaction

Our data reveal interesting findings regarding discrepancy indexes. In terms of differences within the couple no significant differences were found in our sample. In the study of Osin et al. (2018) including couples dealing with a hemato-oncological disease significant differences occurred. The partners showed significantly smaller congruence index, which indicates that the partners could estimate the coping behaviors of the patients more correctly than the other way around. Additionally, the similarity and the perceived similarity indexes suggest that patients and partners reported quite similar estimations of the own coping behavior, but underestimated the other’s coping (Osin et al., 2018). Moreover, Osin et al. (2018) did not differentiate between male and female partners and patients. A possible reason for these differences not appearing in couples coping with kidney transplantation might be that a kidney transplantation does not disturb a couple in their perception as much as a hemato-oncological disease. Patients and partners perceive their coping behavior in the same degree not depending on the role (patient versus partner) when dealing with kidney transplantation. In the sample of a healthy population of Gmelch et al. (2007) these differences did not occur either, which conforms to our assumption.

As expected, almost all discrepancy indexes correlated negatively with psychological outcomes such as DC or partnership quality in both groups, couples with male and female kidney transplant recipients. Low discrepancies were associated with positive psychological outcomes. That is in line with the study of Gmelch et al. (2007).

Our results differ in the group with female kidney transplant recipients from the one of couples with male kidney transplant recipients in regard to depression and anxiety. In couples with male kidney transplant recipients higher discrepancies were associated with negative psychological outcomes, which is in line with Osin et al. (2018). In couples with female kidney transplant recipients negative correlations between discrepancy indexes and psychological outcomes appeared. That means that more congruence correlates with higher levels of depression and anxiety of female kidney transplant recipients. The perception of female kidney transplant recipients of high partner’s coping efforts might lead to feelings of guilt and evoke other negative psychological outcomes like depression and anxiety. The higher depression score of female kidney recipients within the couple supports this assumption. Similar correlation appeared in the study of Osin et al. (2018) where only the small discrepancy within the congruence index was related to high psychological burden. In our data these associations appeared in couples with female transplant recipients within all of the three discrepancy indexes. That might be explained by the issue that female kidney transplant recipients in our sample feel even guiltier because of the dependence on their partner than when patients deal with a hemato-oncological disease. Cancer is perceived as a severe and stressful life event, so that the feelings of dependence and high support from the partner are accepted (Edwards and Clarke, 2004). Contrary, kidney transplantation is seen as a step toward healing and independence (Johnson et al., 1998; Kimmel, 2001). The deception after realizing that one still feels dependent and ill (because of the immunosuppressive medication and permanent confrontation with the foreign organ) might be visible through the discrepancy indexes.

The discrepancy indexes point at female kidney transplant recipients being under higher psychological burden when estimating more correctly the coping efforts of the partner. Discrepancy indexes can help detecting feelings of guilt and might allow analysis of unconscious perception within the dyadic system. To prove all these assumptions more studies analyzing the role of discrepancy indexes are needed.

The relationship quality seems to be one of the most important aspects for the well-being and psychological outcomes of kidney transplant recipients (Frazier et al., 1995). The regression analysis of male kidney transplant recipients in regard to their relationship quality has shown actor effects, the DC total score and the similarity index of the self-evaluation, as the most predictive factors. The DCI total score as the central predictive element of partnership quality is in line with several studies (Gmelch and Bodenmann, 2007). The quality of the DC depends significantly and substantially on the partnership satisfaction, predicted the occurrence of divorce, psychological well-being and psychological disturbances (Kimmel et al., 2000). The second predictive factor was the similarity index of the self-evaluation. In the studies of Gmelch and Bodenmann (2007) and Rohmann and Bierhoff (2007) the perceived similarity index emerged as the most valuable predictive element of relationship quality. The Equity Theory of Walster et al. (1973) supports that the similarity between one another is positively predictive. Interestingly, partner effects of the female partner failed statistical significance. More research is needed to determine the role of several discrepancy indexes, their differences in sex and cut off values that permit the determination of a high/low index. No substantiated cut off values have been determined to our knowledge so far.

The current study has several limitations. The cross-sectional nature of our study does not permit the establishment of casual inferences about the data. Longitudinal designs are needed to prove the validity of the study. Additionally, the study relied on self-reported-perception, which also has to be taken into consideration, as a risk to receive socially desired answers deforming the reality. Nevertheless the analysis of the data showed moderate or significant correlations between patients and partners, so that the reliability of the data is given. The number of dyads with only 20 couples of female transplant recipients was small. Due to the small sample size, the generalizability of the results is limited. To investigate the role of sex larger samples are necessary. Consequently we are inapt to compare the influence of sex on kidney transplant recipients, caregivers, differences in perceived relationship quality or negative psychological outcomes. Longitudinal data are necessary.

Nevertheless, the current study reveals the importance of DC for relationship functioning within kidney transplantation. Contrary to our expectation, female kidney transplant recipients and not female caregivers seem to be the group under risk for developing negative psychological consequences, such as depression. Female kidney transplant recipients seem to profit extensively from the DC. Discrepancy indexes support these assumptions. They appear as an element that could be used in future to reveal more unconscious perceptions within the dyadic interplay. Feelings of guilt or perception of imbalance of a dyad might be detected. Thus the dyadic system of a couple would be reinforced. More research on discrepancy indexes is needed. The findings of the current study could be specifically addressed in interventions for couples with kidney transplantation – especially for couples with female transplant recipients. Strengthening couples’ DC could be a viable option in clinical practice. The dyadic system should be intensified in practice and used as an important way of support within kidney transplantation.

## Author Contributions

TZ and MS have been involved in the development of the design. DT, LF, LP, and MS did the data collection. DT, LF, LP, and TZ have been involved in data analysis and the preparation of the manuscript. All authors contributed to the interpretation of the data. All the authors have read and approved the final manuscript.

## Conflict of Interest Statement

The authors declare that the research was conducted in the absence of any commercial or financial relationships that could be construed as a potential conflict of interest.
